# Product News

**DOI:** 10.1155/S1463924681000448

**Published:** 1981


### Product News

**Editor's Note:** *The address of the manufacturer/supplier appears in italics at the end of each item. In some cases this address will be that of a subsidiary to the manufacturing company as the address given is that from which the information has been obtained.*

### Computer controlled GC

Varian has introduced the GC 6000 microcomputer controlled gas chromatograph featuring an interactive CRT/keyboard for easier operation. It can be coupled to the company's GC 6500 satellite unit, effectively doubling its chromatographic capabilities while saving bench space and capital cost.

The internal memory will store up to eight analysis methods with chromatographic conditions constantly displayed during an analysis.

The GC 6500 satellite facility is controlled from the GC 6000 or a Vista 44; interfacing to the Vista 601 data system is possible to give a powerful automatic unit. Complete automation is possible using Varian auto samplers. A choice of injectors and detectors for packed as well as capillary columns is available, including fused silica.

*Varian Associates Ltd, 24-28 Manor Rd, Walton-on-Thames, Surrey*

### Graphite furnace accessory for AA

Perkin-Elmer's Zeeman/5000 is being introduced as an accessory to the computer controlled Model 5000 atomic absorption spectrophotometer. It combines the HGA-500 furnace with a new approach to Zeeman effect atomic absorption spectrophotometry called transverse AC Zeeman. This feature corrects for background absorption at very high levels (1.5 A and higher) with no loss in sensitivity, and is thus very useful when determining trace elements in oils, biological matrices and steels. The accessory can be easily switched from this mode into double-beam flame atomic absorption with UV/vis background correction if needed.

*Perkin-Elmer Ltd, Post Office Lane, Beaconsfield, Bucks.*

### Ether substitute

Methyl tert-butyl ether (MTBE) which may be used as a direct (and less hazardous) substitute for ethyl ether is now available from Fisher Scientific. The boiling point of MTBE is higher than ether at 55°C and it does not form peroxides as readily. It is available in three purity grades; an HPLC grade, a Certified grade and a straight Purified grade.

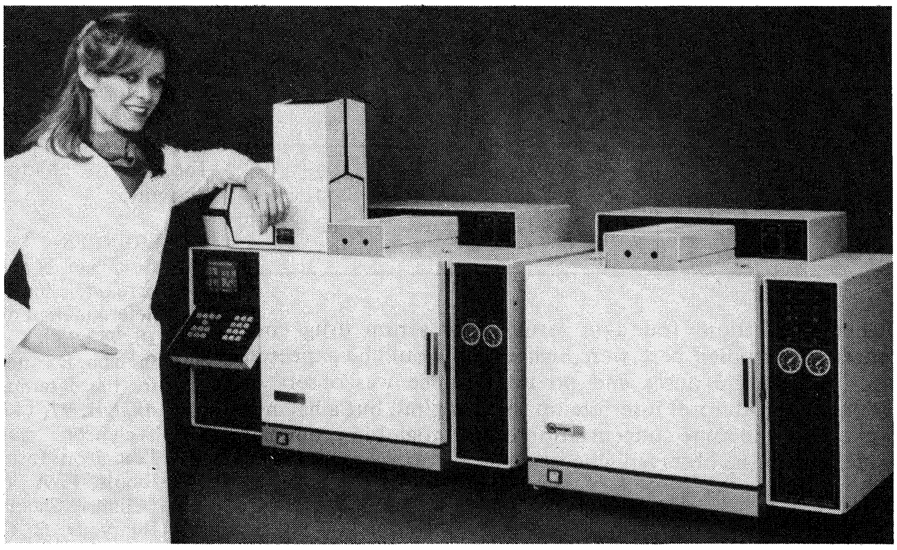

*Varian GC6000 microcomputer controlled gas chromatograph*

### Superbrain!

Anaspec has introduced the Superbrain, a microcomputer intended for scientific and engineering applications with a built-in display, keyboard and floppy disk drive. It's based on the CP/M operating system and allows users to write compiled programs in FORTRAN, APL, COBOL and BASIC. It has 64K of user memory and up to 700K of disk capacity. Hard disks can be added at a later stage to give up to 96 M bytes of storage. Input/output facilities include RS232C and S-100 connections for simplified connections of peripherals, interfaces etc.

*Anaspec Research Laboratories Ltd, Pearl House, Bartholomew St, Newbury, Berks.*

### LC systems

Based on the recently introduced SP 8700 solvent delivery system, Spectra-Physics has announced several new chromatography systems to cover applications for the most basic up to high performance systems. At the bottom of the range, the SP 8700 is combined with the SP 9750 organiser module which links directly with an appropriate detector/recorder or integrator to give flow rates from 0.1 to 10 ml/min for mobile phases of up to three solvents for isocratic, binary gradient or ternary gradient work. The system provides continuous solvent degassing and can sort up to 10 sets of operating conditions entered on the 8700's keypad.

There are also three complete systems covering both fixed and variable wavelength detection, this time based on an SP 8700/8750 combination.

*Spectra-Physics Ltd, 17 Brick Knoll Park, St. Albans, Herts.*

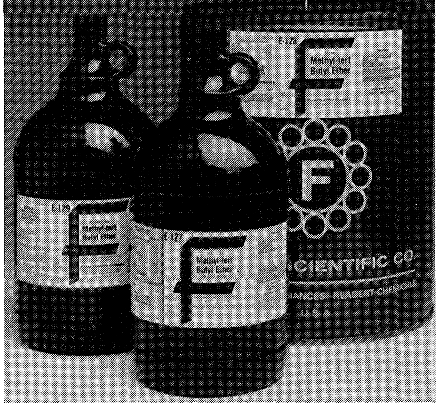

*Fisher Scientific's solvent MTBE*

### Reagent/wash valve

ChemLab's Mk 2 reagent/wash valve is intended to reduce the amount of time it takes to wash through a continuous flow analysis system at the end of a period of analysis. It is said to operate for long periods with minimal attention. Each valve has eight precision, glass-filled PTFE modules fitted with the inlet and outlet nipples for one reagent line and sealed off from the module next to it. A maximum of eight reagent lines can be used.

*ChemLab Instruments Ltd, Hornminster House, 129 Upminster Rd, Hornchurch, Essex RM11 3XJ. Tel Hornchurch 57011*

### Thermal analysis

Mettler's new TA 3000 automated thermal analysis system is built around a microprocessor control centre to give an instrument with programme control of experiment and data analysis from a simple keyboard. The TC10 central processor can be used with differential scanning calorimetry, thermogravimetry or thermomechanical analysis measuring modules and incorporates a range of data evaluation programmes for each module.

*MSE Scientific Instruments, Manor Royal, Crawley, Sussex.*

### Gas chromatograph

Despite the advantages of microprocessor control for your gas chromatograph, the incorporation of sophisticated electronics has often put such instruments out of the reach of many laboratories. To bridge this gap, Packard has introduced the 437 and 438 gas chromatographs which use the established 427 and 428 instruments as their basis with microprocessor control though the company says that this is not achieved at the expense of the price tag. Operation parameters are combined in one fixed programming sequence with LEDs in the input keys to guide the operator through the program.

The 437 is intended for use in routine analyses while the 438 has increased analytical flexibility to make it more suited to complex applications. Five detectors are available; FID, TCD, ECD, NPD and FPD, any two of which can be installed and operated simultaneously. The thermal conductivity detector is a new microcell type with improved lower detection limits.

*Packard Instrument Ltd, 13-17 Church Rd, Caversham, Berks RG4 7AA*

### Null detector

Levell Electronics' null detector type TM10 has two logarithmic ranges. On the mV log range, ± 10% fsd equals ± 5$\mu$V approx, ± 50% fsd equals ± 5 mV approx and ± 100% fsd equals ± 500 mV approx. This range enables null detection to be made throughout an 80 dB change of voltage level without range switching. Signal amplification is by a low drift monolithic integrated circuit controlled by stabiliser circuits to ensure that variations in the supply battery voltage and temperature have minimal effect on performance.

Linear ranges of from ± 30 $\mu$V to ± 300 V and 30 ± pA to ± 300 mA are also covered on the meter.

Battery life is typically over six months.

*Levell Electronics Ltd, Moxon St, Barnet, Herts. Tel 01 449 5028*

### 'Modular' GC System

Pye Unicam is adding to its range of gas chromatographic equipment with six new instruments. The PU4500 series is said to combine high performance and throughput with simple operation and to bring gas chromatography within reach of a wider range of laboratories. System options can employ one or more PU4500s together with other options in the new range such as a microprocessor controlled autojector, a head-space analyser and a video control centre giving multi-channel integration and data handling facilities.

*Pye Unicam Ltd, York St, Cambridge.*

### Luminescence spectrometers

Perkin-Elmer has introduced the LS series of luminescence spectrometers which combine a pulsed xenon source with microprocessor electronics to give a versatile range of instruments. The LS-5 for example measures fluorescence, phosphorescence and chemiluminescence, with a quantum corrected reference system giving fully corrected excitation spectra. The LS-3 is a basic fluorescence spectrometer designed for routine quality control measurements. Semi-corrected excitation spectra are obtained through the use of a microprocessor controlled background corrector.

*Perkin-Elmer Ltd, Post Office Lane, Beaconsfield, Bucks. Tel 04946 6161*

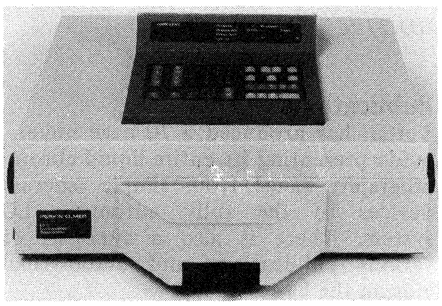

*Perkin-Elmer's LS-5 spectrometer*

### Automated metals analysis by atomic absorption

Varian has introduced two series of atomic absorption spectrophotometer together with the software and a programmable sample changer to give an automated system for metals analysis. The AA 1275/1475 series of AA spectrophotometers has a built-in 8-bit microcomputer for data collection, system management and direct read-out of metal concentrations. The 1275 is a single beam instrument with flame emission capability while the 1475 is double-beamed with flame emission employing a time-shared double-beam system for source drift compensation.

*Varian Associates Ltd, 14-18 Manor Rd, Walton-on-Thames, Surrey.*

### GC gas filter system

Chrompack has introduced a new system for cleaning gas streams in gas chromatography. The design of the filtering systems allows saturated filters to be changed without interrupting the analysis for more than a few seconds. The filter has two elements - the filter itself and a connecting unit. This is equipped with in-and outlet points for the gas flow and two needle valves start and stop the gas flow. Gas flow is started simply by pressing the filter onto the base plate. The filters themselves are made of heavy wall acrylic plastic and contain a high capacity oxygen filter and a moisture trap. There is also a charcoal trap to remove organic compounds. The connecting unit is provided with two ¼ inch male connectors to incorporate the filter in a ¼ inch gas line. An adapter is available if the filters are to be used with ⅛ inch gas lines.

*Chrompack, P O Box 3, 4330 AA Middelburg, The Netherlands*

*Chrompack UK, 61 Shrubbery Rd, London SW16*

### Computer controlled ELISA reader

Artek Systems has launched a fully computer controlled ELISA reader unit, said to be the first of its kind available in Europe. The set-up incorporates a 'dumb' photometer (Model 210) together with a separate computer module (Model 200). The ELISA reader operates under the control of the computer and is said to be compatible with most ELISA kits currently on the market and to accommodate plates with flat, U or V bottom wells. Reading of each 96 well plate (dual blank, negative, low positive, high positive and 44 dual samples per plate) is initiated by inserting the plate into the reader and pressing one button. The ELISA reader has been designed to be adaptable to both turbidimetric and fluorometric assays.

*Artek Systems Corporation, 32 Humberstone Rd, Cambridge, CB4 1JF*

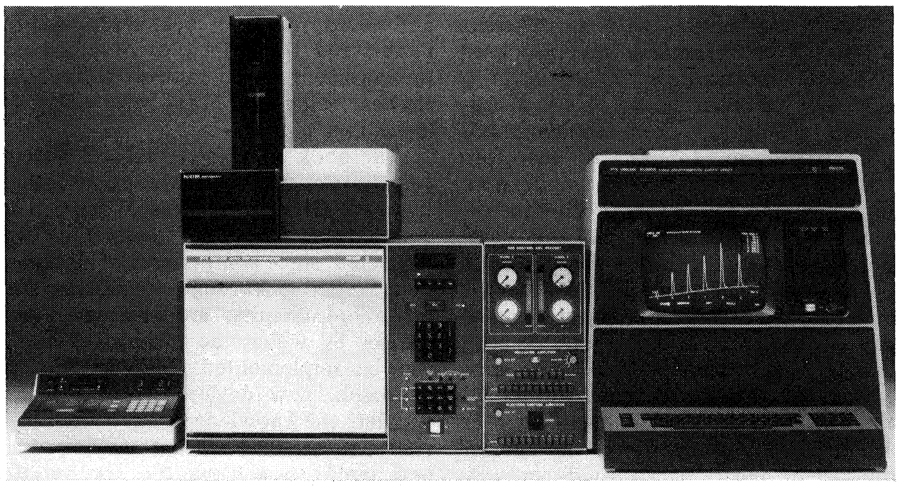

*Pye Unicam's PU4800 video control centre with Series 304 gas chromatograph*

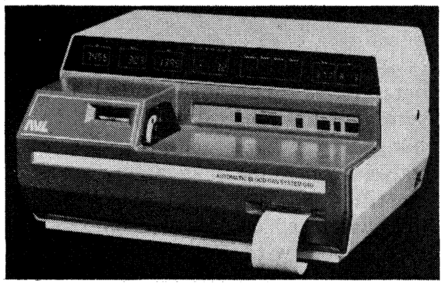

*The AVL 940 automatic blood gas analyser from Laboratory Impex*

### Small sample blood gas analyser

A fully automated pH/blood gas analyser working on 40 μl samples is now available in the UK from Laboratory Impex. The AVL 940 produces digital and hard copy records of $PO_2$, $PCO_2$ and pH. $PO_2$ and $PCO_2$ concentrations are displayed in mmHg, or a readout in kPa is available. The instrument then requires type of patient (adult or foetal), patient temperature and Hb level to provide the following calculated values: base excess *in vivo* and *in vitro*, buffer base, actual bicarbonate, total $CO_2$, oxygen saturation and oxygen content. Full analysis takes 50 seconds plus 15 seconds for presentation of results.
*Laboratory Impex, Lion Rd, Twickenham, Middx. Tel 01 892 9157*

### Recording data processor

Shimadzu has introduced a computerised data processor for use with GC, LC, TLC and AA. The machine features continuously variable peak width integration, full battery protected memory and alpha numeric capability. Up to 339 peaks can be stored and processed to provide data identifications using either absolute retention time, relative retention time, time band or time window methods. Quantitation methods such as area normalisation, corrected area normalisation, scale factors, internal standards and absolute calibration curve are available and concentrations can be presented in any unit of percent, mg, μg and ppm.
*Shimadzu Scientific Instruments Inc, 9147-H Red Branch Rd, Columbia, MD 21045, USA*

### Publications

Varian has produced a 70 page buyers' guide presenting its entire **liquid chromatography** range from simple isocrate devices to the fully automatic LC system. There is also a synopsis of various publications and audio-visual aids on the basics of LC.
*Varian Associates Ltd, 24-28 Manor Rd, Walton-on-Thames, Surrey*

The second quarter, 1981 issue of Technicon's New Products Newsletter, has information on the company's updated product lines, including the new GT system for **accelerated throughput on the AutoAnalyser II**, introduced at Pittsburg, and the AutoAnalyser IIC Plus system for data handling and instrument control.
*Technicon Industrial Systems, Tarrytown, New York 10591 USA*

From Techmation comes a catalogue describing laboratory, on-line and field instrumentation for the analysis and monitoring of principal **air and water pollutants** and physical measurements. Selected products from ten manufacturers are presented.
*Techmation Ltd, 58 Edgware Way, Edgware, Middx. Tel 01 958 3111*

The complete range of Camag **TLC instruments** is presented in a 48 page brochure now available from the company. All phases of TLC process are covered from sample application up to quantitative (densitometric) evaluation. Detail descriptions of apparatus are accompanied by methodological explanations.
*Available from any Camag distributor or agent*

### Book Review

### Electronics and instrumentation for scientists

*Howard V. Malmstadt, Christie G. Enke and Stanley R. Crouch*
*The Benjamin/Cummings Publishing Company Inc, USA*
*Order code: 36917. Price £24.95*

This is the latest edition of what has become one of the standard works for the instruction of scientists, and particularly chemists, in basic electronics and instrumentation. The aim of the book is to provide scientists with an efficient means of understanding the principles of modern electronic instrumentation so that they can confidently choose instruments and apply them to their full potential. The subject is therefore modern electronics with an emphasis on its applications in scientific instrument and control systems and computers. In all its editions the book has been written particularly for students and practitioners in all areas of science and engineering. The presentation is both practical and fundamental. The new edition of the book reflects the fact that we have now entered a new era in scientific instrumentation; the era of microelectronics. Microcircuits provide both the basis and the need for a new approach to the study of electronics. From the users point of view, the availability of complete high performance electronic functions such as operational amplifiers and decade counters in encapsulated, unalterable integrated circuit form, makes the detailed study of transistor amplifier and flip-flop design less critical than a knowledge of the functions performed by the IC's and their applications. At the same time the widespread application of sophisticated electronic functions such as phase-locked loops, analog multipliers and microprocessors creates the need to understand the principles of advanced measurement and control techniques that are being used in the most common laboratory instruments.

The book is organised around system principles and electronic functions rather than the detailed analysis of particular components. The latter however are not neglected, since components of all types, from resistors through transistors and integrated circuits, are studied as the devices by which the various functions can be implemented. Because of this approach, new devices will not make obsolete the knowledge gained from this study; rather they will be recognised as new ways to achieve the appropriate functions. Analog and digital techniques are integrated through the concept of electrical data domains, which are the ways in which data can be encoded in electrical signals. Instrumentation concepts including feedback control, signal-to-noise enhancement, microcomputer interfacing, data acquisition and signal processing are presented along with the techniques and devices for their implementation.

The book as in its earlier editions is useful for structured coursework or for self study. The fourteen chapters included are of nearly uniform length and complexity so that each comprises a similar unit of study. The problems at the end of each chapter are useful for review. This book is organised to support a parallel laboratory if structured coursework is concerned. A list of suggested experiments follows each chapter to indicate the areas of practice the chapter has introduced.

The authors are to be congratulated on the new edition of this standard work; the high standard of production seen in earlier editions has been maintained and its value has been increased immeasurably by the revisions made to include more of the microelectronics which are pertinent to modern analytical instrumentation.

*G.F. Kirkbright*

